# *Rickettsia felis* in Fleas, Southern Ethiopia, 2010

**DOI:** 10.3201/eid1808.111243

**Published:** 2012-08

**Authors:** Oleg Mediannikov, Alemseged Abdissa, Georges Diatta, Jean-François Trape, Didier Raoult

**Affiliations:** Institut de Recherche pour le Développement, Dakar, Senegal (O. Mediannikov, G. Diatta, J.-F. Trape);; Jimma University, Jimma, Ethiopia (A. Abdissa);; and Aix Marseille University, Faculté de Médecine, Marseille, France (D. Raoult)

**Keywords:** Rickettsia felis, flea-borne, fleaborne spotted fever, Ethiopia, rickettsioses, flea, Ctenocephalides felis, Pulex irritans, bacteria, Rickettsia, parasites

**To the Editor:** Fleas (order Siphonaptera) are obligate hematophagous insects. They are laterally flattened, holometabolous, and wingless ectoparasites. More than 2,500 species of flea, belonging to 16 families and 238 genera, have been described. A minority of these genera live in close association with humans (synanthropic), including fleas of these species: *Pulex irritans*, *Ctenocephalides felis*, *Ctenocephalides canis*, *Xenopsylla cheopis*, *Nosopsyllus fasciatus*, *Echidnophaga gallinacea*, and *Tunga penetrans* (*1*). Many fleas are capable of transmitting the following pathogens to their hosts: bacteria (e.g., *Rickettsia typhi, R. felis, Yersinia pestis,* and many *Bartonella* spp.); viruses (e.g., myxoma virus); protozoa (e.g., *Trypanosoma* spp.); or helminths (e.g., *Hymenolepis* spp.) (*2*). *Ctenocephalides* spp. fleas are of special interest as main reservoirs and vectors of *R. felis*, because this agent causes an emerging disease, fleaborne rickettsiosis. The distribution and prevalence of this disease have not been well studied. Symptoms of this disease range from mild to moderate and include fever, cutaneous rash, and sometimes an inoculation eschar (*3**,**4*). *R. felis* can also infect at least 10 other species of arthropods, including *P. irritans* fleas*,* trombiculid and mesostygmata mites, hard and soft ticks, and booklice (*5**,**6*).

In Africa, the presence of *R. felis* in fleas has been documented in Algeria, Tunisia, Egypt, Ethiopia, Gabon, Côte d’Ivoire, and the Democratic Republic of Congo (*5*). Recent studies conducted in Senegal (*3*) and Kenya (*4*) have shown that as much as 4.4% and 3.7%, respectively, of acute febrile diseases in these regions may be caused by *R. felis* infections. We conducted a study to determine the distribution and prevalence of *R. felis* in fleas in Ethiopia.

In our study, 55 fleas were collected in 2010 in 2 villages in Ethiopia; 25 fleas were collected from Tikemit Eshet (6°51′837″N and 35°51′348″E; altitude2,121 m), and 30 fleas were collected from Mizan Teferi (6°59′640″N and 35°35′507″E; altitude 1,700 m). The specimens were collected by using a plate filled with soapy water with a candle in the middle of the plate. Because fleas are thermotropic, they jumped toward the candle and fell onto the plate, where they rapidly drowned in the soapy water. The fleas were identified by morphologic features and stored in 90% ethanol until DNA extraction.

To confirm the phenotypic identification, we designed primers and probes for quantitative real-time PCR (qPCR) that were specific for 2 species of flea (*P. irritans* and *C. felis*) based on the sequences of mitochondrial cytochrome oxidase gene published in GenBank (Table). All of the identifications made by morphologic appearance were confirmed by qPCR because some specimens were damaged and difficult to identify. We found that most (52/55) of the fleas collected in human dwellings were *P. irritans*, and 3 specimens were *C. felis*. A screening by amplification using primers and probes specific for the 16S–23S internal transcribed spacer of *Bartonella* spp (*7*). produced no positive results.

**Table:** Sequences of primers and probes used to identify fleas by quantitative real-time PCR, southern Ethiopia, 2010

Species	Forward primer, 3′→5′	Reverse primer, 3′→5′	Fluorescent probe
*P. irritans*	CGAATACTTTTAGAAAGCCAAAACA	CATTGATGACCAATAGATTTTAGAGTG	TTGCTTTACCGTCTTTACGTTT
*C. felis*	TCGTTATTTACTTGAAAGACAAAATG	TCATTGATGACCAATTGCTTT	TGCTTTACCTTCTCTTCGACTTTT

*P., Pulex; C., Ctenocephalides.

We screened rickettsial DNA by using qPCR with a *Rickettsia*-specific, *gltA* gene-based RKND03 system (*8*) and a *bioB*-based qPCR system specific for *R. felis*. We found that the 3 specimens of *C. felis* fleas contained the DNA of *R. felis*; however, 23 (43%) of 53 *P. irritans* specimens also contained DNA of *R. felis*. We amplified and sequenced nearly the entire rickettsial *gltA* gene from 3 *C. felis* and 10 *P. irritans* specimens and found that the sequence was identical to that of *R. felis* (GenBank accession no. NC_007111).

During the field collection of the fleas, the conservation of specimens may be difficult. Degradation of specimens may pose a problem for the ensuing morphologic identification. For fleas, a specific preparation is required that destroys internal organs and produces a chitin complex of the insect. This type of preparation makes it difficult, and sometimes impossible, to use the insect later for molecular studies. The development of qPCR specific for *P. irritans* and *C. felis* fleas facilitated the identification of damaged samples and also precluded the laborious and time-consuming procedure of identification by morphologic features.

We conclude that the reservoirs of *R. felis* in Ethiopia include both *C. felis* and *P. irritans* fleas. In Ethiopia, *P. irritans* fleas have been reported to be prevalent (*9*). *P. irritans* fleas have been shown to be infected by *R. felis* in several locations, notably in the Democratic Republic of the Congo and in the United States, and another rickettsia phylogenetically similar to *R. felis* has been detected in *P. irritans* fleas in Hungary (*10*). Reports attributing substantial numbers of acute febrile illnesses to fleaborne rickettsiosis caused by *R. felis* in Senegal and Kenya (*3**,**4*) place fleaborne rickettsiosis among emerging diseases with the potential for adverse public health effects. Furthermore, the identification of the vectors of *R. felis* in Ethiopia reveals the epidemiologic background for the fleaborne spotted fever in this region. We speculate that the elucidation of the full range of possible vectors of *R. felis* may facilitate the development of prevention measures that will help control this disease.
